# OLDER WOMEN AND HIV: THE IMPACT OF PROVIDER COMMUNICATION OF HIV/AIDS ON CONVERSATIONS WITH NEW SEXUAL PARTNERS

**DOI:** 10.1093/geroni/igad104.0448

**Published:** 2023-12-21

**Authors:** Erin Robinson, Geunhye Park, Getrude Nyang’au

**Affiliations:** University of Missouri School of Social Work, Columbia, Missouri, United States; University of Missouri/ University of Illinois-Chicago, Chicago, Illinois, United States; University of Missouri - Columbia, Columbia, Missouri, United States

## Abstract

Older adults account for 50% of people living with HIV and nearly 20% of new HIV infections in the U.S. Inadequate communication with a healthcare provider has been identified as a contributing factor, as stigma surrounding older adults, sex, and HIV/AIDS is persistent. Many older adults report having conversations with their healthcare providers about HIV prevention more routinely in their younger years. To date, however, little is known about provider communication of HIV with older patients. This cross-sectional study aims to help fill that gap. Survey participants (N=427) were asked about the frequency in which their primary healthcare providers talked with them about HIV/AIDS before they turned 50, as well as their future likelihood to discuss HIV/AIDS with a new sexual partner. Participants ranged in age from 50 to 84 years (M=58; SD=6.3), and were predominately white (94%) and female (73%). Regression analysis demonstrated that among women in the sample, provider communication about HIV/AIDS prior to them turning 50 year of age was associated with their future likelihood of talking with new sexual partners about HIV/AIDS (β=0.151, p<.05). Eighty-seven percent of the sample reported intentions to discuss condoms with a new sexual partner with a mean of 4.89 (SD=1.47, Range=1-6) indicating “Somewhat Agree” and “Agree”. These findings suggest that provider communication of HIV/AIDS is important for risk prevention. Those conversations help guide future decision making among women in particular, even into their later years.

